# A neonatal intensive care nurse experience of an automated chyme reinfusion therapy device: A case report describing use in a neonate with necrotising enterocolitis

**DOI:** 10.1016/j.intf.2025.100038

**Published:** 2025-01-28

**Authors:** Taylor Harrington, Emma Ludlow

**Affiliations:** a Auckland City Hospital, New Zealand; b University of Auckland, New Zealand; c The Insides Company, New Zealand

**Keywords:** Distal feeding, mucous fistula refeeding, double enterostomy, feed intolerance, prematurity, NEC

## Abstract

**Background:**

Neonatal intestinal failure related to double enterostomies is rare and associated with high morbidity and mortality. Chyme reinfusion therapy (CRT) remediates high outputs and improves nutrition and electrolyte balance of neonates. Low adoption of CRT due to the messy administration has constrained use of this beneficial therapy. An automated CRT system is now available to improve nursing workflow.

**Case report:**

This case report explores a neonatal intensive care nurse experience of managing a neonate using CRT with The Insides® Neo (The Insides Company, New Zealand). Their parents learnt to use the system with their child while they were in the neonatal intensive care unit (NICU). Nursing workflow improved along with the parents only requiring a short training period to manage the system. Dramatic weight gain and cessation of parenteral nutrition were achieved.

**Conclusion:**

The automated CRT system refined the administration of CRT allowing for improved nursing workflows, increased parental contact with their child, and ameliorated clinical outcomes.

## Introduction

Intestinal Failure is an inability to sustain life due to inadequate intestinal function, necessitating nutritional and fluid support [1]. Enterostomy formation in neonates and children can lead to intestinal failure which has significant morbidity and mortality [2]. Surgically treated necrotising enterocolitis (NEC) is the main cause of enterostomies in the neonatal population, especially in extremely low birth weight (ELBW) infants. The incidence of NEC in ELBW ranges from 2 % to 13 % [3]. Symptoms like abdominal distension and inadequate feeding are initially managed medically, however 27–52 % of patients deteriorate, requiring surgical intervention [3].

Management involves a multidisciplinary approach to ensure adequate nutrition, address high outputs, manage enterostomies, and provide psychological support to families [4]. Enteral autonomy is preferred to mitigate risks from parenteral nutrition, including line sepsis and metabolic issues [5]. Achieving enteral autonomy may involve gastric administration of expressed breast milk (EBM) or enteral nutrition via orogastric or nasogastric tubes, along with chyme reinfusion therapy (CRT) to maximize nutrient absorption [4], [6].

CRT is the reintroduction of a patient's own chyme that is transferred down the distal limb of their small intestine double barrel enterostomy or enterocutaneous fistula to allow further absorption of nutrients and fluid. CRT offers benefits such as improved growth, intestinal maturation, and restoration of liver and renal function, though its use has been limited by equipment challenges and inefficient hospital consumables [4], [7]. Systematic reviews in 2020 and 2022 emphasized the need for a standardized CRT delivery method to reduce adverse events [4], [8], [9]. An automated CRT system, The Insides Neo, was developed to address these issues. A ten-patient feasibility study showed improved nursing workflows and positive clinical outcomes [10]. This case report details a neonatal intensive care nurse's experience with the device in a neonatal intensive care unit (NICU).

## Automated CRT system

**Fig. 1:**
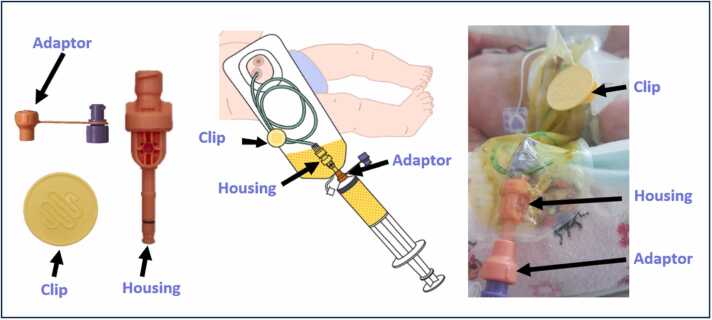
Diagram of the components of The Insides Neo. (*Photo reproduced with family consent*).

**Fig. 2:**
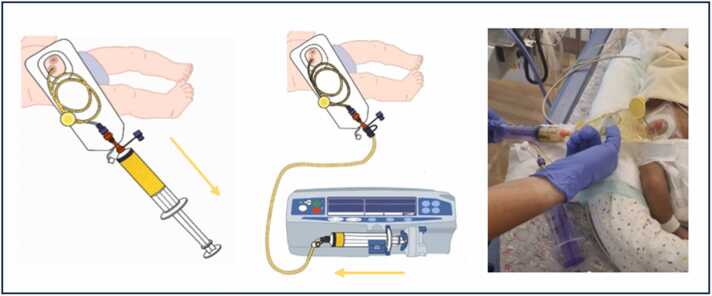
Diagram of performing continuous chyme reinfusion therapy with the automated CRT system (*Photo reproduced with family consent*).

The automated CRT system is a directional flow accessory for standard neonatal stoma appliances. As shown in Fig. 1, the housing fits into the stoma appliance's aperture and connects to a ENFit™ feeding tube. The adaptor is externally secured with a push-twist-click motion. After setting up the stoma appliance and feeding tube in the distal enterostomy, a clip is attached to secure the distal feeding tube, externally. The device allows for bolus or continuous reinfusion. Continuous reinfusion is performed by withdrawing chyme into an ENFit™ syringe every 4 hours and reinfusing over the following 4 hours using a syringe pump (Fig. 2).

## Patient history

Simon (pseudonym) was born at 24 + 1 week’s gestation, weighing 650 g and was initially in good condition. He went to NICU on continuous positive airway pressure (CPAP), received one dose of surfactant, and was treated with caffeine for apnoea of prematurity. He was given antibiotics for 36 hours, which were stopped after negative blood cultures.

**Fig. 3:**
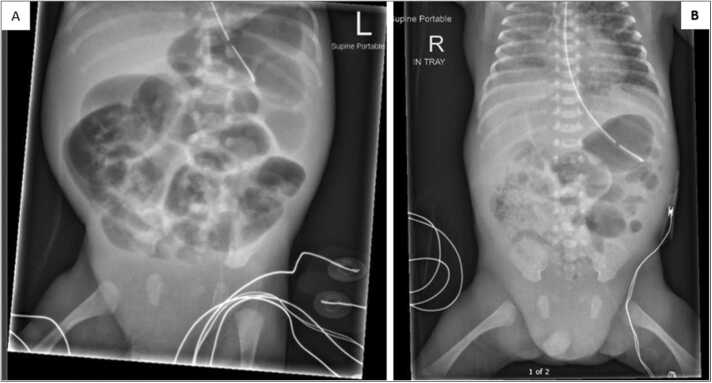
Simon’s abdominal x-ray demonstrating aerophagia (A) and perforation (B). (*Photo reproduced with family consent*).

By day of life (DOL) 7, Simon was tolerating 120 ml/kg/day of fortified expressed breast milk (EBM) via orogastric. Simon had normal stools and a soft abdomen. However, on DOL 8, he deteriorated with marked abdominal distention, feed intolerance, and increase in apnoeic episodes. Escalation protocol was followed, and he was intubated due to a profound apnoeic episode and was exhibiting signs of NEC with presumed sepsis. He was placed NBM and an abdominal x-ray revealing findings consistent with Aerophagia, sometimes known as ‘CPAP belly’ (Fig. 3**A**). Aerophagia is where a portion of air that should be directed to the lungs, inadvertently makes its way into the GI tract. The symptoms of aerophagia mirror NEC and a diagnosis can generally only be made via abdominal x-ray.

Despite initial monitoring, Simon deteriorated further. By DOL 10, x-rays indicated an asymmetrical mottled gas pattern and significantly dilated small bowel. On DOL 12, x-rays revealed abnormal gas patterns, bowel wall thickening, interloop fluid, and free air in the abdomen, indicating perforation (Fig. 3**B**). At 25 + 6 weeks corrected gestation, weighing 800 g, he underwent surgery to resect 10 cm of necrotic ascending colon and the ileocaecal valve, resulting in a double enterostomy. The proximal small bowel remained intact with the proximal limb of the enterostomy brought out at the terminal ileum and the mucous fistula (distal limb) originating from the distal ascending colon.

## Post-operative

Simon faced a challenging post-operative recovery, experiencing acute kidney injury due to hypotension and requiring inotropic support. He had significant laparotomy wound dehiscence necessitating special dressing changes and prolonged ventilation support, with multiple failed extubation attempts. On post-operative day (POD) 36, he was successfully extubated to CPAP after dexamethasone treatment at corrected gestational age of 29 + 2.

Simon dealt with high enterostomy output averaging 40–50 mls/kg/day, relying on parenteral nutrition (PN) and electrolyte infusions. From POD 2 to day 41, he gained only 50 g in total, with his growth chart plateauing and shifting toward the 3rd centile for his corrected gestation. Despite multidisciplinary team efforts to increase gastric feeds and introduce a fortifier, enterostomy output increased dramatically, necessitating a return to trophic gastric feeds and increased PN requirements.

To control his high output, efficiently manage his nutrition and fluid balance, and better support his growth, Simon was considered for CRT and worked up accordingly. Once his laparotomy dehiscence had mostly healed and the integrity of the distal bowel was assessed via contrast imaging, the automated CRT device was commenced. Simon’s enterostomy and mucous fistula outlets were able to be contained in a neonatal stoma appliance.

## Device training, set-up, and stabilisation

NICU nurses received comprehensive training on the device through a combination of instructional videos, formal education sessions, hands-on cot-side teaching, and a resource document kept at the cot-side containing key information to support use.

At baseline, Simon’s enterostomy outputs were high at 45 ml/kg/day, receiving gastric feeds of EBM at 3 ml/hour and 200 ml/kg/day of parenteral nutrition. Simon was started on the automated CRT device at 31 + 5 weeks corrected gestation (DOL 53), POD 41, weighing 1150 g. The cot-side nurse assembled the device with a size 6 Fr gastric tube for refeeding, inserted into the distal fistula and advanced to the 5 cm marking. The first and second assembly of the device by each of the cot-side nurses took approximately 5 minutes but reduced with increased experience.

Due to historical CRT experience, the initial protocol involved starting with half the emptied volume of chyme every four hours for the first 24 hours. Simon passed 10 ml of chyme every 4 hours so 5 ml of chyme was reinfused over the following 4 hours at a rate of 1.25 ml/hour for the first 24 hours. This approach allowed the nursing team to monitor for signs of reflux from the distal lumen and maintain Simon’s comfort considering he had significant enterostomy outputs. He progressed to full volume reinfusions from 24 hours (10 ml output in 4 hours that was reinfused at 2.5 ml/hour over the following 4 hours). The surgical team stopped intravenous replacement of enterostomy losses the same day due to him tolerating reinfusion of all his output at 2.5 ml/hour with no reflux. Simon had his first bowel movement within 48 hours of starting CRT.

Stoma appliance changes were needed every 3 days due to leaks underneath the appliance baseplate however, the skin under the appliance remained intact. When the stoma appliance (assembled with the device) was changed in the first week, some cot-side nurses were finding it difficult to identify the proximal and distal lumen. This resulted in a few instances where the proximal lumen was cannulated by mistake resulting in perceived higher stoma outputs and ineffective CRT. A patient ‘Cot Card’ was developed that clearly displayed the location of the proximal/distal anatomy, tube size, and maximum reinfusion rate. Once the ‘Cot Card’ was introduced there were no further incidences of incorrect placement of distal tubing.

**Fig. 4:**
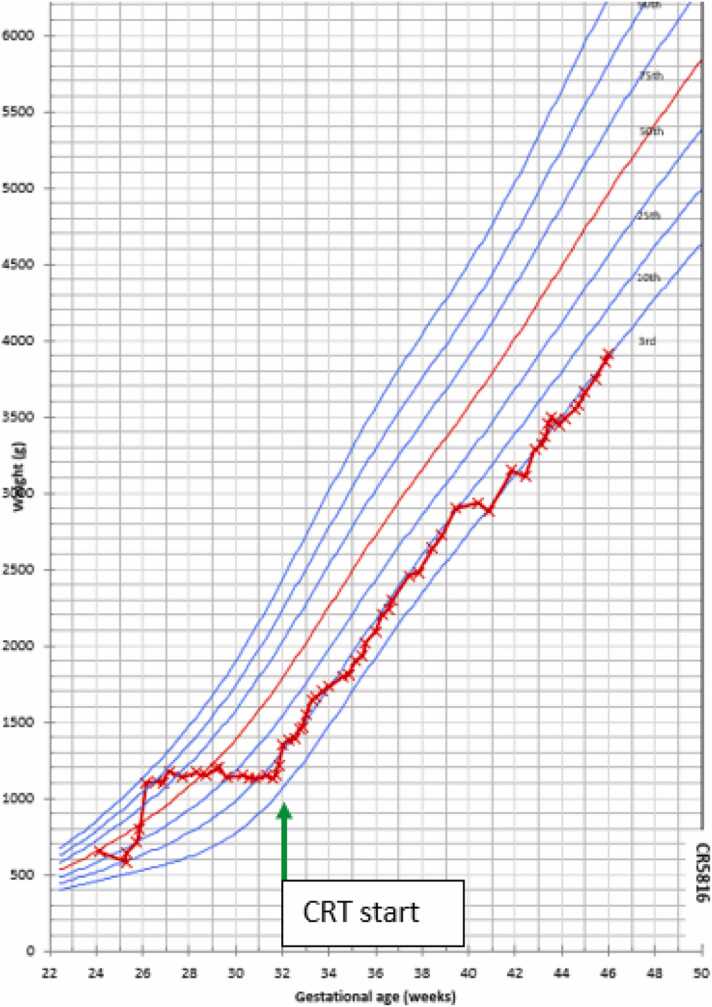
Simon’s Fenton growth chart.

Simon was able to tolerate the introduction of fortified EBM gastric feeds which were progressively increased and in tandem, his PN volume reduced, reaching full gastric feeds of fortified EBM of 180 ml/kg/day in 14 days. His growth chart normalised, tracking along the 10th centile (Fig. 4.). At corrected gestational age 33 + 5 (DOL 67), he weighed 1700 g, gaining 550 g (40 g/day) in 14 days which provided the impetus to wean from PN and have his central line removed.

## Parent training, re-anastomosis, and recovery

Once Simon was stabilised on CRT, his parents requested to learn how to manage Simon’s care. Having watched the cot-side nurses, they quickly became proficient in all aspects of his refeeding care within 14 days (one month after commencing the device). They were trained with similar training material to the nursing team and then supported to manage device assembly, stoma appliance changes, tube insertion, chyme withdrawal, and continuous reinfusion. Simon developed a distal limb prolapse which brought his re-anastomosis forward. On DOL 111, 40 weeks corrected, weighing 2900 g, Simon went back to surgery for his re-anastomosis. He spent a total of 58 days on the device and gained a total of 1750 g (average daily weight gain 30 g/day). He had a routine post-operative recovery where he remained nil-by-mouth for six days to give his anastomosis time to heal, and then was slowly titrated back up to full enteral feeds in three days.

## Discussion

This case report demonstrates the successful use of an automated CRT device to support a neonates nutrition and improvement in weight gain and clinical outcomes. The device learning curve for the NICU cot-side nurses was approximately 5 days and allowed Simon’s parents to manage the device for him due to its ease of use. Nutrition is one of the cornerstones of neonatal care, optimising and accelerating growth and weight goals is even more pivotal in the neonate with a high output enterostomy due to their reduced intestinal capacity for absorption [3], [4], [7].

Manual CRT was the standard of care prior to the introduction of the automated CRT device in this NICU. Manual CRT posed significant challenges for the cot-side nursing staff, particularly around maintaining the stoma appliance's integrity, dislodgment of feeding tubes multiple times a day, and leakage at the puncture site necessary for tube insertion. This leakage, along with the need for regular stoma appliance changes, often led to compromised peristomal skin integrity and frequent excoriation, complicating patient care [4]. Their experience motivated their eagerness to trial a device aimed at addressing these complications. The transition to automated CRT likely benefited from familiarity, enabling them to appreciate the device's advantages. Feedback from the cot-side nurses caring for Simon indicated notable improvements in workflow efficiency (<5 min set-up). Cot-side nurses found the device intuitive, collectively reporting confidence in its use after just a few cycles reinfusion. They preferred this automated method over the manual approach for its cleanliness and ease.

Simon’s parents only experienced the device (with no prior knowledge of manual CRT) but they commented on the apparent ease for nursing staff which is why they requested to learn. This aided in fostering bonding opportunities for the family in the heavily medicalised setting of a NICU. Empowering a family to manage part of their child's care encourages further involvement and reassures their ability to manage their care once discharged home [11]. A systematic review completed on the effect of family centred care in a NICU setting concluded that increasing opportunities for parents to bond, increases positive patient outcomes overall [11].

Simon struggled to make significant weight gain prior to commencing CRT. The efficacy of PN in promoting optimal growth in neonates, particularly preterm infants, remains a complex and incompletely understood area [12], [13]. While PN serves as a critical lifeline for neonates unable to tolerate enteral feeding, achieving adequate growth with PN alone presents significant challenges [12]. This difficulty is likely multifaceted, encompassing impaired neonatal metabolic processes, elevated and compounding energy expenditure relative to nutrient intake, and the inherent limitations of PN formulations in fully replicating the complex nutritional profile of breast milk [12]. Clinicians recognise this challenge, as evidenced by the widespread practice of supplementing PN with enteral nutrition as soon as feasible, along with the increased attention paid to careful monitoring of growth parameters and adjustments to PN composition. Despite the development and implementation of PN guidelines aimed at optimising nutrient delivery, evidence continues to support enteral nutrition accelerate growth with utilisation of the entire intestine for absorption via CRT [4], [8], [12], [13].

A limitation of the device is that it currently fits with neonatal stoma appliances and once the neonates enterostomy exceeds the template of the baseplate, the device is unable to be used. This issue arose when Simon’s distal limb prolapsed and he required a larger template. A prolapse is generally regarded as a good sign that there are minimal adhesions and it is safe to move forward with re-anastomosis [1], [9]. In spite of this, his accelerated growth meant he was nearing the template limitations of the neonatal stoma appliance and concerns arose among nursing staff and his parents regarding a potential transition back to manual CRT with a different stoma appliance which was not looked upon favourably. This underscores the need for continuing innovation in device design to meet the evolving requirements of neonatal patients.

## Conclusion

CRT using The Insides® Neo is shown to be safe and effective at improving weight gain and improving clinical outcomes in this case report. The device learning curve for the cot-side NICU nurse was 5 days and was positively received by the wider NICU nursing team due to the ease of use and optimised workflows. The neonate’s parents were able to manage his device care providing increased bonding opportunities in a NICU setting.

## Ethical clearance

Ethical clearance was not required.

## Funding

This case report did not receive funding.

## Patient Consent

Simon is a pseudonym. Parental consent has been provided to share his story and use clinical images.

## CRediT authorship contribution statement

**Harrington Taylor:** Writing – original draft, Conceptualization. **Ludlow Emma:** Writing – review & editing.

## Declaration of Competing Interest

Taylor Harrington contracts to The Insides Company. Emma Ludlow is an employee of The Insides Company.
